# An examination of the complex climate risks and management approaches confronting the Northeast Tiger and Leopard National Park in the future

**DOI:** 10.1038/s41598-026-52830-y

**Published:** 2026-05-26

**Authors:** Yan Wu, Xuan Liu, Zhiqiang Zhao

**Affiliations:** 1 College of Landscape Architecture, Northeast Forestry University, No. 26, Hexing Road, Xiang Fang District, Harbin, 150040 Heilongjiang China; 2 Faculty of Architecture and Design, Harbin Institute of Technology, Harbin, 150006 China; 3 Prioritized Laboratory of National Territory Spatial Planning and Ecological Restoration in Cold Regions, Ministry of Natural Resources, Harbin, 150006 China; 4 Harbin Municipal Government Investment Project Service Center, Harbin, 150077 China

**Keywords:** CMIP6 downscaling, Biodiversity conservation, Climate adaptation, Adaptive management, Amur tiger, Climate sciences, Ecology, Ecology, Environmental sciences, Hydrology, Natural hazards

## Abstract

The Northeast Tiger and Leopard National Park is crucial for global biodiversity protection; yet, its environment is under considerable threat from climate change. The absence of a comprehensive grasp of future risk parameters by area managers may impede the development of effective adaptation solutions. This study utilized a “contrasting futures” climatic concept to tackle the issue of “decision uncertainty”. A hybrid statistical downscaling method was employed to downscale CMIP6 multi-model data to 0.1°, selecting two extreme scenarios under the SSP5-8.5 framework: “dry-hot” (EC-Earth3-Veg) and “warm-wet” (MIROC6). The research evaluated two bias correction techniques, quantile mapping (QDM) and random forest (RF), selecting the superior method to produce a high-confidence dataset for the analysis of extreme occurrences. The forecasts indicate that the park will undergo warming in both scenarios, yet the nature and dimensions of the threats vary: the “dry-hot” scenario suggests a substantial rise in temperature, with drought leading to habitat degradation and heightened wildfire risks; the “warm-wet” scenario intensifies ecological corridor disruption and geological disasters resulting from extreme precipitation. The analysis indicates that the regional effects of global climate change impacting the park present a twin threat of compounded drought and flood. The findings offer essential direction for enduring adaptive management and policy structures, highlighting the ineffectiveness of singular catastrophe response approaches and promoting versatile tactics that accommodate several scenarios to safeguard this prominent region.

## Introduction

The IPCC AR6 Synthesis Report states that global temperatures have increased by 1.1 °C^1^, heightening the possibility of climate tipping points. Should temperatures exceed these limits, internal amplification phenomena, such as permafrost thawing or extensive forest dieback, may transpire^2,3^. Furthermore, pertinent research indicates that the frequency of climate anomalies in Northeast China has markedly risen^4^. The Northeast Tiger and Leopard National Park serves as a premier site for the conservation of temperate forest ecosystems in China and is essential for safeguarding endangered species like the Siberian tiger and Far Eastern leopard. The core objective of China’s national park policies is to protect these vital natural ecosystems, a mission that deeply aligns with the United Nations Sustainable Development Goals (SDGs), particularly SDG 13 (Climate Action) and SDG 15 (Life on Land). As a crucial ecological space, the park not only conserves biodiversity but also reduces carbon emissions, acting as a robust ecological barrier to mitigate climate change impacts. Addressing its management complexities requires the incorporation of Environmental, Social, and Governance (ESG) frameworks to provide a sustainable basis for resource utilization. By integrating SDGs into national park policies, this region can provide a valuable Chinese solution” for global ecological governance and international cooperation.

Global climate change exacerbates regional vulnerabilities within the park, yet managers face significant decision-making uncertainties when devising adaptive strategies. While the CMIP6 dataset provides the foundation for climate forecasting^5–10^, its direct application is hindered by coarse resolutions and systematic biases^11^. Traditional methods for assessing climate impacts rely on multi-model ensembles (MME)^12^ or reliability ensemble averaging (REA) approaches^13^. However, these methods are aimed at identifying the most likely central tendency^14^. During the ensemble process, variance is smoothed, and the outcomes of extreme climate events in predictions are weakened. In ecological risk management, relying on such ensemble averages can lead to underestimation of reasonable, high-impact risks. Addressing this uncertainty^13,15^ requires a shift in approach: moving from attempting to predict a single possible future to preparing for a range of plausible scenario conditions^16^. Inspired by advanced climate scenario planning frameworks, this study adopts a contrasting futures approach. By deliberately selecting the most divergent climate predictions, a comprehensive uncertainty envelope is established. This method provides managers with a robust stress-testing tool^17^, ensuring that conservation strategies remain viable under all potential climate extremes, rather than just under the mathematical average.

In order to apply future scenario approaches at the specific scale of national parks, this study uses a hybrid statistical downscaling technique, combining Delta bias correction and the Random Forest (RF) algorithm to achieve a high-resolution grid of 0.1°. This method can effectively capture the long-term trends of Global Climate Models (GCMs) while resolving complex nonlinear local environmental interactions^18,19^. From the CMIP6 ensemble, we selected two boundary scenarios under the SSP5-8.5 pathway: one dry-hot future (EC-Earth3-Veg) and one warm-wet future (MIROC6). Since ecological tipping points, such as compound droughts and wildfires, are usually triggered by daily extreme events rather than monthly averages, rigorous bias correction and preservation of extreme events at the daily scale are crucial. This study systematically evaluates the performance of the Quantile Delta Mapping (QDM) and Random Forest (RF) in preserving the statistical characteristics of historical extreme events, ultimately selecting the method with better performance to generate high-fidelity datasets for dynamic risk assessment^20^.

The main objectives of this study are threefold: (1) to develop two types of future extreme climate scenario data for the Northeast Tiger and Leopard National Park; (2) to quantify the trends and occurrence frequencies of management-critical compound extreme events, such as severe droughts, floods, and corridor risks; (3) to translate these quantitative risk assessments into forward-looking, climate-smart spatial management strategies.

## Materials and methods

**Fig. 1:**
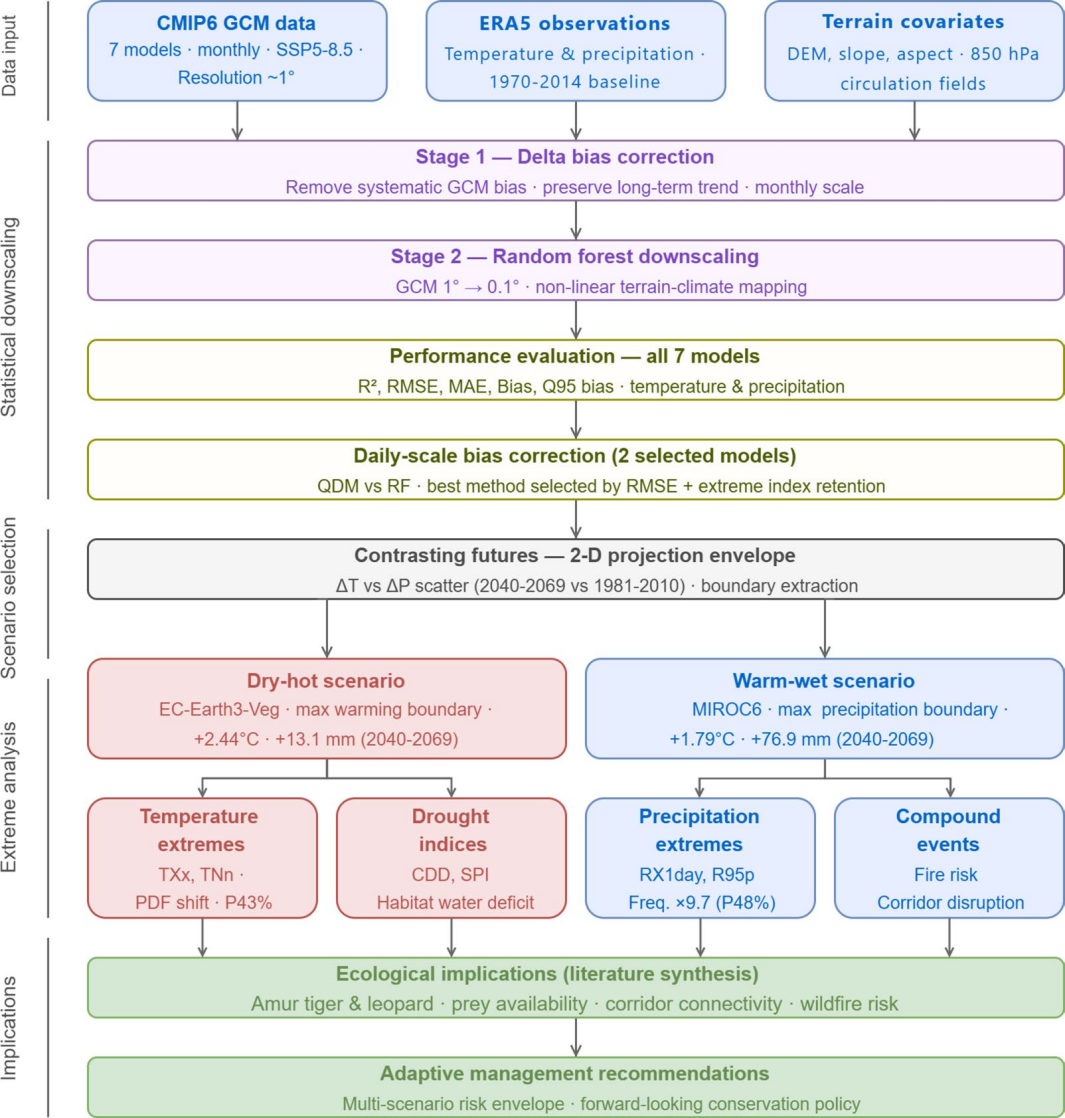
Workflow Diagram.

The overall methodological workflow of this study is presented in Fig. 1, encompassing data input, two-stage downscaling, model selection, extreme event analysis, and ecological risk assessment.

### Study area

The Northeast China Tiger and Leopard National Park is located between 42° 31′ 06″ N–44° 14′ 49″ N and 129° 5′ 0″ E–130° 18′ 48″ E, covering an area of 14,926 km^2^^21^. The area is mostly defined by mid- to low-elevation mountains and hills, with elevations under 1600 m. The park’s forested regions contain multiple rivers with short valleys, creating north–south biological corridors. The climate is categorized as temperate continental monsoon, including annual mean temperatures between 2.1 and 5.7 °C and significant seasonal fluctuations, annual precipitation amounts to 600–800 mm, with around 60% occurring in the summer months (June–August)^22^. This study expands the park’s limits by extracting a 0.1° spatial buffer to assure the completeness of climate grid data and ecological consistency in frontier regions. Thus, the study area includes segments of exterior ecological buffer zones and regions of human activity. This inclusive geographical delineation method enables a thorough evaluation of regional climatic attributes while reducing potential border impacts on climate analyses.

**Fig. 2:**
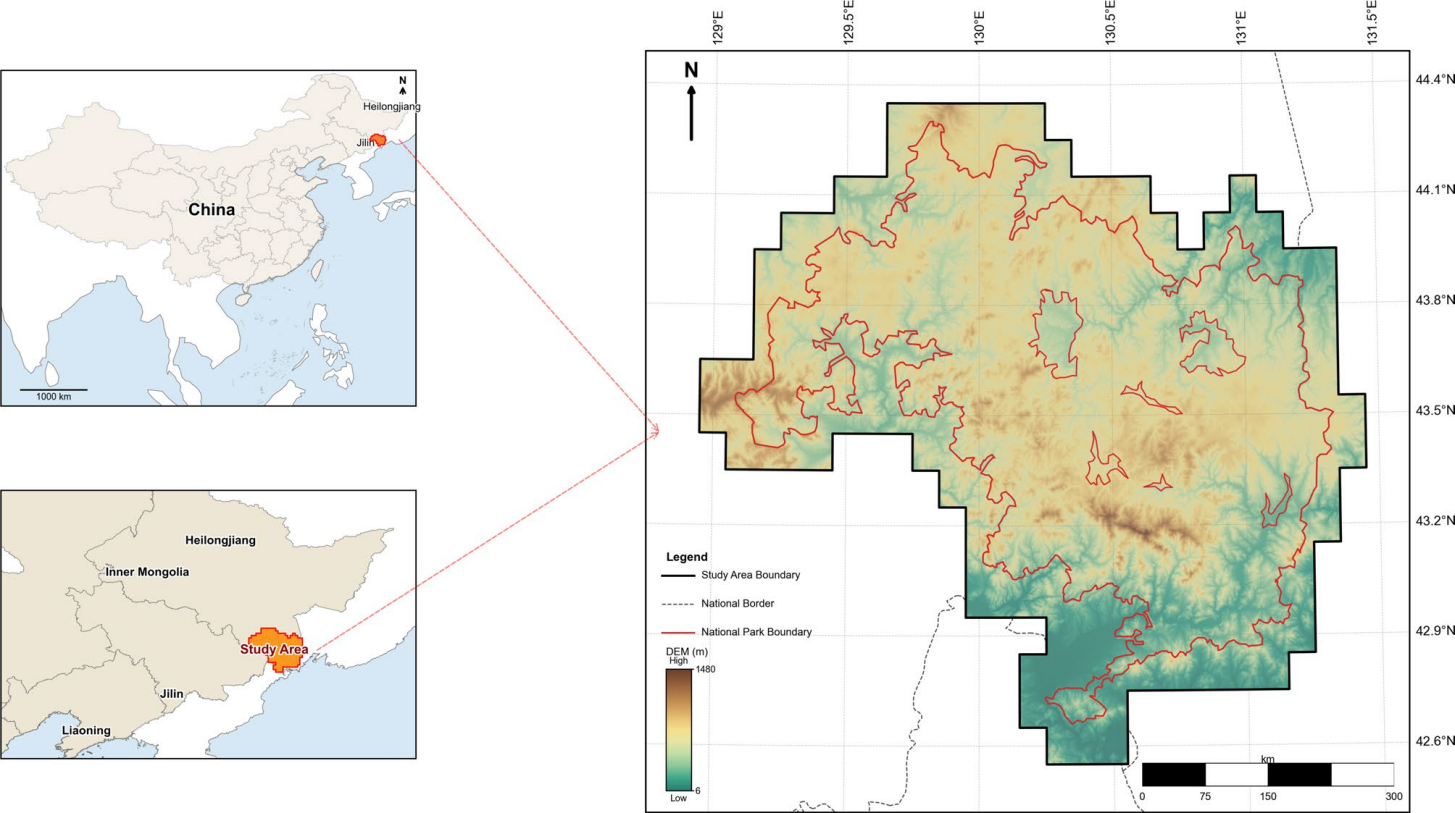
Study area location and topographic characteristics.

Figure 2 illustrates the geographical location, elevation gradient, and ecological corridor distribution of the study area. Map created by the authors using Python (v3.9.13) with Cartopy and Matplotlib. DEM: NASA SRTM via Google Earth Engine^23^. National park boundary: General Plan for the Northeast Tiger and Leopard National Park (2023–2030). Provincial boundaries: Ministry of Natural Resources of China (Map Approval No. GS(2019)1822).

**Fig. 3:**
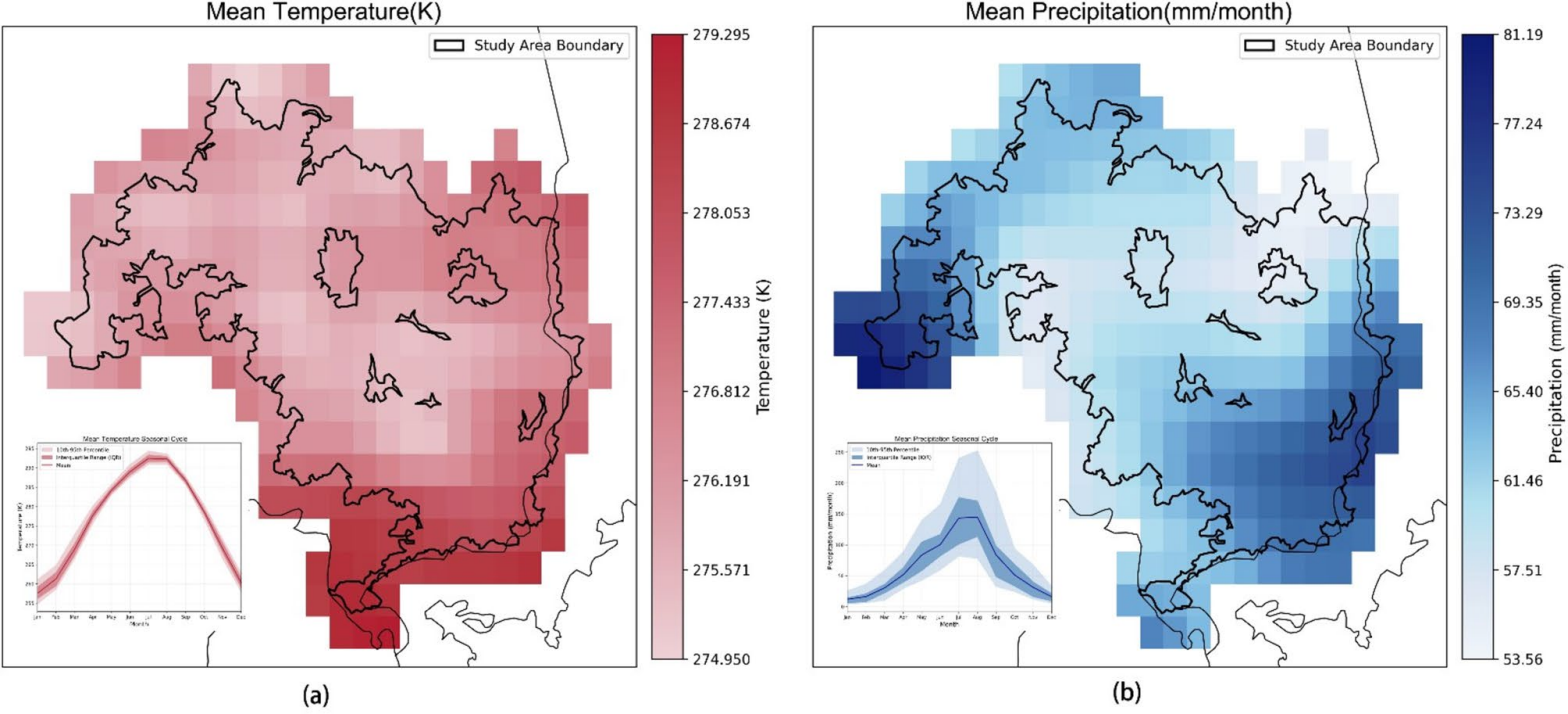
Spatial and seasonal variations of temperature and precipitation from 1970 to 2024.

As shown in Fig. 3, during the period from 1970 to 2024, the multi-year mean temperature in the study area ranges from 274.95 to 279.29 K (Fig. 3a), while precipitation ranges from 53.56 to 81.18 mm/month (Fig. 3b). Both temperature and precipitation exhibit pronounced seasonality, with concurrent hot and humid conditions in summer. The 10th–90th percentile range for precipitation is largest in July–August, indicating the greatest interannual variability in midsummer precipitation and a heightened susceptibility to extreme flood or drought events.

### Data description

Observational data were obtained from the ERA5-Land global land surface reanalysis dataset, published by the European Centre for Medium-Range Weather Forecasts (ECMWF) inside the Copernicus Climate Change Service (C3S) framework (https://cds.climate.copernicus.eu). This dataset attains a spatial resolution of 0.1° × 0.1° (about 9 km), exceeding the 0.25° resolution of the original ERA5 atmospheric reanalysis dataset. The ERA5-Land dataset facilitates the precise characterization of land surface climate changes in localized areas through its hourly-scale outputs^24^. ERA5-Land, with temporal coverage from 1950 to the present, offers a solid basis for long-term, high-accuracy regional climate assessments in this research. The main variables obtained from ERA5-Land consist of 2-m air temperature (2 m T) and total precipitation (TP). To improve the physical realism of precipitation downscaling, supplementary variables from the ERA5 dataset were integrated: specific humidity (q), the eastward wind component (U-component of wind, u), and the northward wind component (V-component of wind, v). These auxiliary variables are crucial for delineating atmospheric moisture transport and dynamical characteristics.

This research employs climate data from seven Global Climate Models (GCMs) included in The Scenario Model Intercomparison Project (ScenarioMIP) is the primary activity within Phase 6 of the Coupled Model Intercomparison Project (CMIP6), under two separate Shared Socioeconomic Pathway (SSP) scenarios: SSP2-4.5 and SSP5-8.5. The data include monthly precipitation and mean temperature, along with daily precipitation and mean temperature. All model data were sourced from the official Earth System Grid Federation (ESGF) data portal (CMIP6-IPSL—Home|ESGF-CoG). Comprehensive model details are provided in Table 1. The chosen scenarios comprise SSP2-4.5, illustrating an intermediate pathway where atmospheric carbon dioxide concentrations stabilize over time due to the implementation of emission-reduction strategies and technologies; and SSP5-8.5, portraying a business-as-usual trajectory characterized by escalating carbon dioxide emissions over time. Only calendars of the Gregorian, standard, and proleptic Gregorian kinds were preserved to maintain uniformity in temporal data scales. The historical baseline period for this study is delineated as 1970–2014, while the future period extends from 2015 to 2100, categorized into the near-term (2015–2039), mid-century (2040–2069), and late-century (2070–2100) segments of the twenty-first century, considering the temporal coverage of both ERA5-Land and CMIP6 datasets. Spatial resolutions were standardized with observational data using bilinear interpolation for temperature variables and cautious interpolation for precipitation variables.

**Table 1:** CMIP6 mode related information

Number	Model name	Institution and country	Resolution
1	EC-Earth3	EC-Earth Consortium, including multiple European institutions（Europe)	1.125° × 1.125°
2	EC-Earth3-CC	EC-Earth Consortium, including multiple European institutions（Europe)	1.125° × 1.125°
3	EC-Earth3-Veg	EC-Earth Consortium, including multiple European institutions（Europe)	1.125° × 1.125°
4	EC-Earth3-Veg-LR	EC-Earth Consortium, including multiple European institutions（Europe）	1.125° × 1.125°
5	MIROC6	Atmosphere and Ocean Research Institute , University of Tokyo; National Institute for Environmental Studies; RIKEN （Japan）	1.4° × 1.4°
6	MRI-ESM2-0	Meteorological Research Institute （Japan）	1.1° × 1.1°
7	NESM3	Nanjing University of Information Science and Technology (China)	2.8° × 2.8°

### Research methods

#### Delta bias correction

The Delta technique essentially modifies mean biases in climatic variables by calculating monthly variances (Delta values) between model simulations and historical observations, based on the premise that these discrepancies remain constant over time. These bias corrections are then implemented on forthcoming climate data, thereby maintaining the variability traits of the original data, along with fluctuations resulting from global land surface processes and global circulation physics parameterizations, facilitating an accurate depiction of future climate alterations^25^. This method rectifies absolute biases while preserving the model’s long-term trends, thereby enabling further modeling endeavors. Delta bias correction is not a downscaling technique akin to machine learning models,instead, it serves as a data preparation phase that provides consistent and interpretable input climate features for subsequent machine learning-based modeling. The mathematical equations utilized in the Delta approach are as follows:

1 $${P}_{\Delta }={P}_{\mathrm{Mod,daily}}{\left(\frac{{\overline{P}}_{\mathrm{obs}}}{{\overline{P}}_{\mathrm{Mod}}}\right)}_{\mathrm{Monthly}}$$

2 $${T}_{\Delta }={T}_{\mathrm{Mod,daily}}+\left({T}_{\mathrm{obs,ymoni}}-{T}_{\mathrm{Mod,ymoni}}\right)$$

${P}_{\Delta },{T}_{\Delta }$ denote the precipitation and monthly temperature adjusted via the Delta method, respectively, ${P}_{\mathrm{Mod,daily}}$, ${T}_{\mathrm{Mod,daily}}$ represent the monthly precipitation and temperature simulated by the GCM, ${\overline{P}}_{\mathrm{obs}}$, ${T}_{\mathrm{obs,ymoni}}$ indicate the long-term monthly mean precipitation and temperature recorded at observational stations, ${\overline{P}}_{\mathrm{Mod}}$, ${T}_{\mathrm{Mod,ymoni}}$ denote the long-term monthly mean precipitation and temperature simulated by the GCM.

#### Random forest (RF) model

While data modified using the Delta adjustment roughly correspond with regional averages, they do not adequately reflect the spatial variety of local climates. This work utilizes a non-parametric machine learning method, specifically the Random Forest (RF) algorithm, as a secondary tool for enhanced statistical correction. Random Forest is a type of decision tree ensemble learning that effectively models nonlinear interactions between climate variables and multivariate predictors. Utilizing Bootstrap Aggregating (Bagging) and Random Subspace Methods to construct multiple decision trees significantly diminishes prediction variance and addresses remaining biases between the first adjusted CMIP6 monthly-scale data and the ERA5-Land reference data^26–28^.

The statistical downscaling of near-surface air temperature (tas) utilizes input features that include diverse terrain-derived variables and temporal encodings, such as digital elevation model (DEM), slope, aspect, topographic position index (TPI), topographic wetness index (TWI), distance to coast, longitude and latitude, and land cover types, to comprehensively represent the influencing effects of topography and geographic location on temperature. Temporal characteristics are highlighted to illustrate seasonality.

Total precipitation (tp) data generally display a pronounced positive skew in their distribution. This work employs a logarithmic transformation to preprocess both CMIP6 model outputs and observed precipitation data, enhancing their distributional features. The intricacy of precipitation stems from its spatial variability and irregularity; thus, input features are classified into three categories: large-scale circulation field variables, surface static variables, and temporal-seasonal factors. Feature engineering integrates relationships between meteorological circulation and topography to elucidate the complex physical determinants of precipitation.

Downscaling outcomes are assessed utilizing metrics including the coefficient of determination (R^2^) and root mean square error (RMSE) (Tables 1 and 2), alongside feature importance rankings conducted for the precipitation model (Table 3). Utilizing the high-precision monthly datasets produced in this phase, the study identifies the two GCMs with the most significant variations in temperature and precipitation for further daily studies.

**Table 2:** Downscaling assessment results for temperature variables

Model name	Type	R^2^	RMSE	MAE	Bias	Relative RMSE	R^2^IR (%)	RMSE IR (%)
EC-Earth3-CC	Raw	0.9348	3.8509	2.9730	–1.8705	1.3922	–	–
	Down	0.9717	2.5346	1.9076	–0.0624	0.9163	56.42	34.21
EC-Earth3-Veg-LR	Raw	0.8767	5.2945	4.1349	–3.5326	1.9140		–
	Down	0.9745	2.4068	1.7487	–0.2616	0.8701	78.96	54.54
EC-Earth3-Veg	Raw	0.9122	4.4662	3.4493	–2.1505	1.6146	–	–
	Down	0.9727	2.4903	1.7435	–0.1934	0.9003	69.77	44.25
EC-Earth3	Raw	0.9300	3.9888	3.0742	−1.8953	1.4420	–	–
	Down	0.9747	2.3999	1.7772	0.0070	0.8676	63.86	39.83
MIROC6	Raw	0.9545	3.2162	2.5251	0.0692	1.1627	–	–
	Down	0.9772	2.2772	1.6638	–0.0892	0.8233	50.00	29.20
MRI-ESM2-0	Raw	0.9488	3.4125	2.6349	–0.9557	1.2337	–	–
	Down	0.9744	2.4127	1.7503	–0.0862	0.8722	49.61	29.32
NESM3	Raw	0.9401	3.6898	2.7130	–1.1727	1.3339	–	–
	Down	0.9742	2.4232	1.8623	0.0596	0.8760	59.96	34.33

The Down indicates “Downscaled”. *IR* stands for improvement rate.

**Table 3:** Downscaling assessment results for total precipitation variables

Model name	Type	R^2^	RMSE	MAE	Bias	Relative RMSE	R^2^IR (%)	RMSE IR (%)
EC-Earth3-CC	Raw	0.1004	58.8931	39.5117	8.6045	88.2662	–	
	Down	0.7175	33.0035	21.8160	–2.9674	49.4641	68.56	43.96
EC-Earth3-Veg-LR	Raw	–0.0048	62.2444	43.2437	9.7686	93.2890	–	
	Down	0.7231	32.6753	21.8931	–3.2217	48.9722	72.31	47.50
EC-Earth3-Veg	Raw	0.1159	58.3862	38.9316	10.3667	87.5066	–	
	Down	0.7147	33.1667	21.8927	–3.0948	49.7088	67.59	43.19
EC-Earth3	Raw	0.1143	58.4390	40.7367	8.5944	87.5858	–	
	Down	0.7131	33.2599	22.1138	–2.8540	49.8484	67.85	43.09
MIROC6	Raw	0.0996	58.9216	40.5392	20.1249	88.3090	–	
	Down	0.7321	32.1398	21.4109	–2.6885	48.1696	70.23	45.45
MRI-ESM2-0	Raw	0.1991	55.5694	36.8233	−4.3709	83.2849	–	
	Down	0.7186	32.9375	21.9574	−3.0534	49.3653	64.83	40.73
NESM3	Raw	0.0785	59.6061	42.5427	17.0446	89.3349	–	
	Down	0.7153	33.1327	22.0312	−3.3148	49.6578	68.56	44.41

The Down indicates “Downscaled”. *IR* stands for improvement rate.

#### Daily-scale data correction

Targeted correction procedures are applied to account for the physical disparities across different climate factors and variations in evaluation outcomes. The approach for temperature variables remains consistent. Nevertheless, due to the significant intermittency, randomness, and highly skewed distributions of precipitation data, the RF approach often underestimates precipitation values. Quantile Delta Mapping (QDM) is employed for bias reduction to guarantee the veracity of extreme event analysis^29^. QDM is a non-parametric, distribution-based correction method that fundamentally aligns the probability distribution function of the adjusted data with that of the reference data (ERA5-Land). This method’s evaluation focuses not on daily coefficients of determination (R^2^), but on its ability to avoid systematic biases and accurately replicate the entire statistical distribution, especially for extreme values. This method conforms to established bias correction approaches employed in the international climate impact assessment framework ISIMIP, therefore producing reliable results.

The study evaluates the efficacy of RF- and QDM-corrected daily-scale precipitation data in retaining extreme events, ultimately affirming QDM’s superiority in tackling extreme precipitation issues within the study area and designating it as the preferred method for producing daily-scale precipitation projection datasets.

### Declaration on the use of generative artificial intelligence (GenAI)

In the preparation of this manuscript, generative artificial intelligence (GenAI) was utilized to assist with language refinement, including enhancements to grammar and spelling, thereby improving the overall clarity and readability of the paper.

## Research results

### Downscaling evaluation results

This offers a comparative table of the evaluation outcomes for data processed via bias correction and random forest modeling. The assessment outcomes are presented in the Table 1 below.

For temperature variables, the raw GCM simulations demonstrate a significant goodness-of-fit, with an average coefficient of determination (R^2^) of 0.9282. After processing with the random forest model, the average R^2^ increases slightly to 0.9742, indicating a rise of 0.046. This advancement signifies that the strategy further refines the association between simulated and observed data while maintaining large-scale climate signals. Metrics include root mean square error (RMSE), mean absolute error (MAE), bias, and relative root mean square error (Relative RMSE) exhibit regular patterns. RMSE scores span from 2.28 to 5.29 (mean: 3.25 ± 0.85), indicating modest error levels in forecasting observed variables. The MAE ratings, averaging 2.48 ± 0.76, further emphasize the model’s precision in representing central patterns. Bias estimates indicate a consistent underestimating in the model’s downscaled results. Feature importance analysis reveals that raw CMIP6 data is predominant (Gini index = 0.58 ± 0.03), succeeded by monthly variables (0.39 ± 0.02), but DEM-related characteristics combined contribute less than 0.1 in significance. Multi-model validation exhibits significant consistency in feature importance rankings across several hyperparameter configurations (Spearman ρ = 0.98), confirming the robustness of the study’s findings.

The evaluation results for precipitation variables, based exclusively on temporal and associated geographic factors, were inadequate. Incorporating supplementary large-scale precipitation-related environmental variables during training significantly enhanced model performance, effectively eradicating first-order systematic biases in the raw model outputs (bias reduced from + 8.6 to + 20.1 to − 3.3 to − 2.9). The coefficient of determination (R^2^) significantly rose from an initial range of 0.0–0.2 to around 0.7, indicating an improvement in variance explanation of 64.8–72.3%. Regarding error control, the root mean square error (RMSE) and mean absolute error (MAE) diminished by 40.7–47.5% and 49.0–50.5%, respectively, while the relative error reduced from 83.3–93.3% to 48.2–49.8%. The enhancements demonstrated uniformity among models with varying physical processes, including EC-Earth3, MIROC6, and MRI-ESM2-0, hence confirming the method’s robustness and generalizability in multi-model scenarios. Feature relevance was determined using the random forest’s mean decrease in impurity technique, with higher values signifying stronger relative contributions to precipitation predictions. Table 3 illustrates that the feature relevance rankings across the seven models exhibit significant ensemble consistency. The integrated humidity index, specific humidity, temperature, moist-enthalpy proxy, and meridional wind sequentially emerge as the most significant predictors.

The five foremost features exhibit consistent hierarchical topologies and relative significance across all models (Table 4). The target variable’s sensitivity to input factors demonstrates considerable robustness, remaining unaffected by substantial variations in underlying physical processes among models. Given the existing training samples and parameter configurations, the random forest method reaches identical optimal splitting strategies, indicating a fundamental and clearly defined discriminative structure inside the data feature space.

**Table 4:** The top five most important features for precipitation variables and their importance

Model name	Humidity index	q	t	qt	v
EC-Earth3	0.1777	0.1368	0.1196	0.1121	0.0609
EC-Earth3-CC	0.1675	0.1408	0.1214	0.1175	0.0592
EC-Earth3-Veg	0.1694	0.1384	0.1254	0.1127	0.0630
EC-Earth3-Veg-LR	0.1700	0.1371	0.1235	0.1154	0.0621
MIROC6	0.1723	0.1364	0.1233	0.1152	0.0620
MRI-ESM2-0	0.1701	0.1416	0.1207	0.1126	0.0640
NESM3	0.1690	0.1361	0.1238	0.1158	0.0627

Humidity Index represents the comprehensive humidity index, q represents specific humidity, t represents temperature, qt represents the combined index of specific humidity and temperature, and v represents meridional wind.

The results indicate that this hybrid downscaling paradigm improves the usability of CMIP6 data at regional levels, thereby offering a more precise data basis for climate impact assessments.

### Model selection

**Fig. 4:**
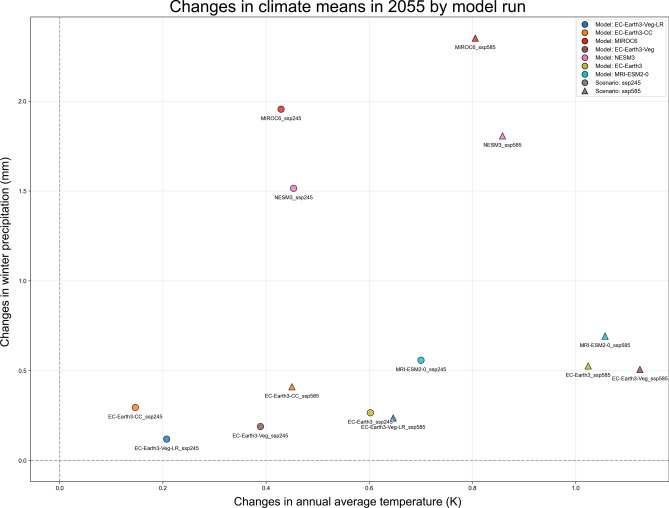
Climate change projections of temperature and precipitation by CMIP6 model and scenario (2055).

Figure 4 and Table 5 depicts the anticipated climate changes in the study area for 2055, compared to the period of 1981–2010, based on multiple climate models, all of which indicate a trend of rising temperatures. Analysis of the distributions of temperature changes (ΔT) reveals ranges from 0.14 to 1.12 K, whilst changes in winter precipitation (ΔP) range from 57 to − 3.58 mm. The research employs the EC-Earth3-Veg model to depict the “hot and dry” climate scenario and the MIROC6 model to illustrate the “warm and wet” climate scenario. The simulation outcomes from these two models under the SSP5-8.5 scenario represent the maximum probable variations in temperature and precipitation for the study area, providing a basis for further severe case evaluations.

**Table 5:** Projected changes in annual mean temperature and winter precipitation by 2055 under different SSP scenarios

Model	Scenario	Temp_change (K)	Precip_change (mm)
EC-Earth3-CC	Ssp245	0.15	0.30
EC-Earth3-CC	Ssp585	0.45	0.41
EC-Earth3-Veg-LR	ssp245	0.21	0.12
EC-Earth3-Veg-LR	Ssp585	0.65	0.24
EC-Earth3-Veg	Ssp245	0.39	0.19
EC-Earth3-Veg	Ssp585	1.12	0.51
EC-Earth3	Ssp245	0.60	0.27
EC-Earth3	Ssp585	1.02	0.53
MIROC6	Ssp245	0.43	1.96
MIROC6	Ssp585	0.81	2.35
MRI-ESM2-0	Ssp245	0.70	0.56
MRI-ESM2-0	Ssp585	1.06	0.69
NESM3	Ssp245	0.45	1.52
NESM3	Ssp585	0.86	1.81

### Performance evaluation of RF and QDM on daily-scale data

The RF model exhibits enhanced performance for daily temperature data, with evaluation findings indicating improvements over the baseline models, facilitating the production of high-precision and dependable daily temperature sequences.

The assessment outcomes of the two approaches for daily precipitation data are displayed in Table 3. In comparison to observational data, the unprocessed outputs from the EC-Earth3-Veg model demonstrate significant discrepancies, with all numerical metrics signifying the inadequacy of the raw GCM data. After applying the QDM approach, the systematic bias in the EC-Earth3-Veg model is substantially mitigated, decreasing the bias to 0.1108 and the RMSE to 2.3354. The RF model enhances R^2^ to 0.4682 and produces a superior RMSE relative to the raw data; it raises the bias to 0.6201, leading to a systematic underestimating overall. This generates more averaged predictions that disregard systemic biases and the attributes of extreme values. Comparable assessment results are noted for the MIROC6 model. RMSE is especially responsive to significant errors caused by extreme events; hence, although QDM’s performance for R^2^ is inferior, it successfully reduces fundamental systematic biases and maintains precipitation extremes.

Table 6 illustrates that for both the EC-Earth3-Veg and MIROC6 models, RF correction significantly enhances the R^2^ values compared to the reference data, increasing from an initial range of around 0.08–0.09 to about 0.47, indicating an improvement rate above 42%. Nonetheless, the efficacy of RF in minimizing mistakes is somewhat restricted, with RMSE enhancement rates ranging from 47 to 53%, and an inconsequential influence on systematic bias (Bias). Conversely, QDM produces only negligible increases in R^2^ values (improvement rate under 1%), however it significantly diminishes model mistakes. The QDM-corrected RMSE for the EC-Earth3-Veg and MIROC6 models reduces by 68.9% and 73.3%, respectively, accompanied by significant reductions in MAE. Significantly, the systemic biases in the raw models have been mitigated, with Bias values lowered by around 80–84%.

**Table 6:** RF and QDM assessment results

Model	Type	R^2^	RMSE	MAE	Bias	R2_IR%	RMSE_IR%
EC-Earth3-Veg	GCM	0.0793	7.5108	3.4026	0.6166	–	–
EC-Earth3-Veg	RF	0.4682	3.9186	1.9802	0.6201	42.24	47.82
EC-Earth3-Veg	QDM	0.0853	2.3354	1.0302	0.1108	0.651	68.90
MIROC6	GCM	0.0929	8.2769	3.8120	0.9490	–	–
MIROC6	RF	0.4745	3.8956	1.9623	0.6048	42.07	52.93
MIROC6	QDM	0.0983	2.2129	0.9669	0.1547	0.595	73.26

*IR* stands for improvement rate.

**Fig. 5:**
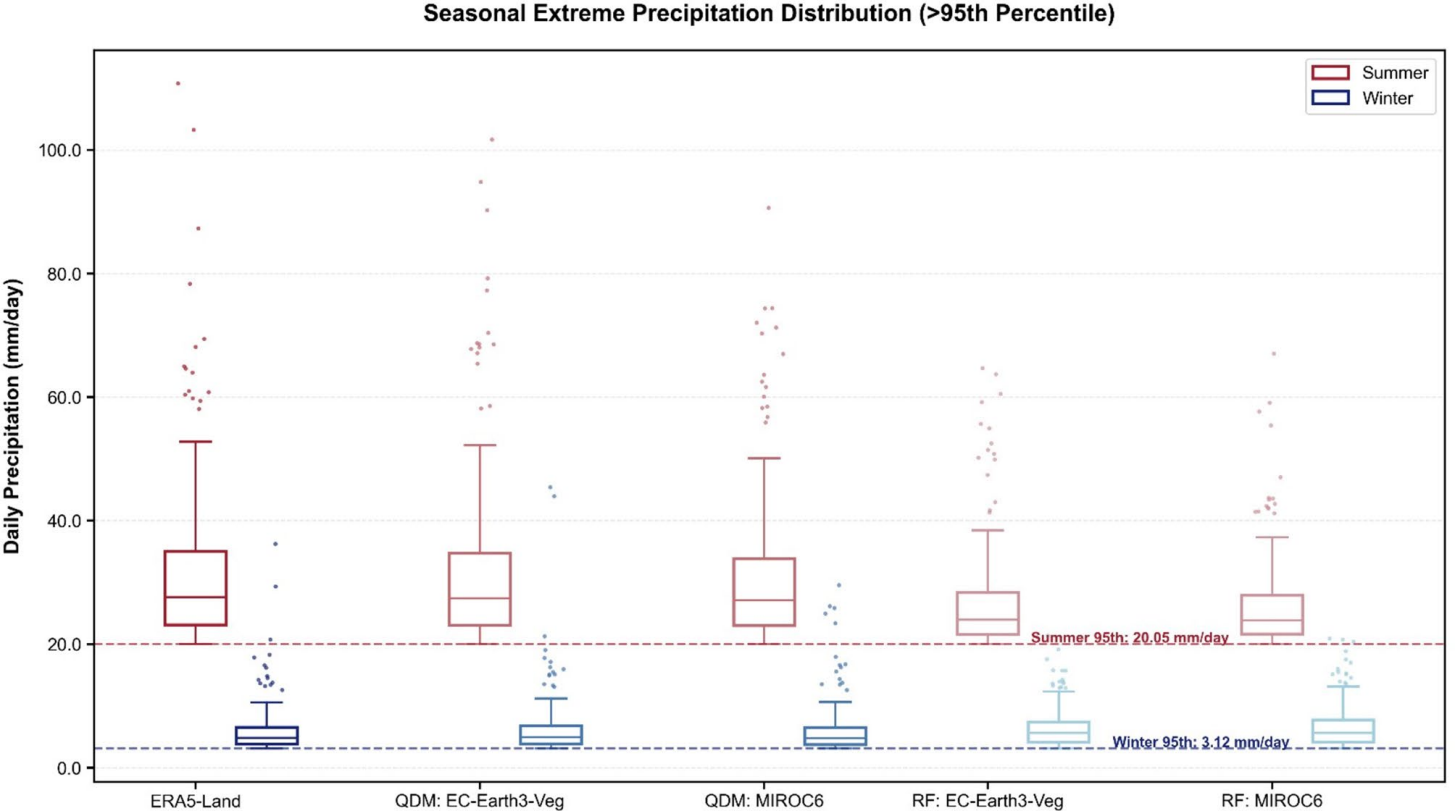
Seasonal extreme precipitation distribution (95th percentile) across bias-correction method.

A comparative analysis of the statistical distributions of daily precipitation amounts surpassing the 95th percentile thresholds from historical reference data (20.05 mm/day for summer and 3.12 mm/day for winter) was conducted to evaluate the efficacy of the two methods in replicating extreme precipitation events, as depicted in Fig. 5. The median values for the QDM-corrected EC-Earth3 and MIROC6 models in summer are 28.14 mm and 27.75 mm, respectively, closely aligning with the reference median of 28.23 mm. Conversely, the RF-downscaled outcomes for the identical models produce medians of 24.35 mm and 24.25 mm, respectively, signifying that QDM attains closer alignment with the reference. Moreover, the RF model significantly constrains the variability space of extreme occurrences, with this “peak-clipping” phenomenon being most pronounced in the higher tail of the distribution. The top whisker endpoints for the RF-corrected EC-Earth3 and MIROC6 models are 41.16 mm and 40.23 mm, respectively, both below the reference value of 57.11 mm.

This study concludes that the extensive benefits of QDM in total quantity control and the replication of extreme events allow it to more effectively maintain and rectify such occurrences.

### Overall trends in future climate changes

**Fig. 6:**
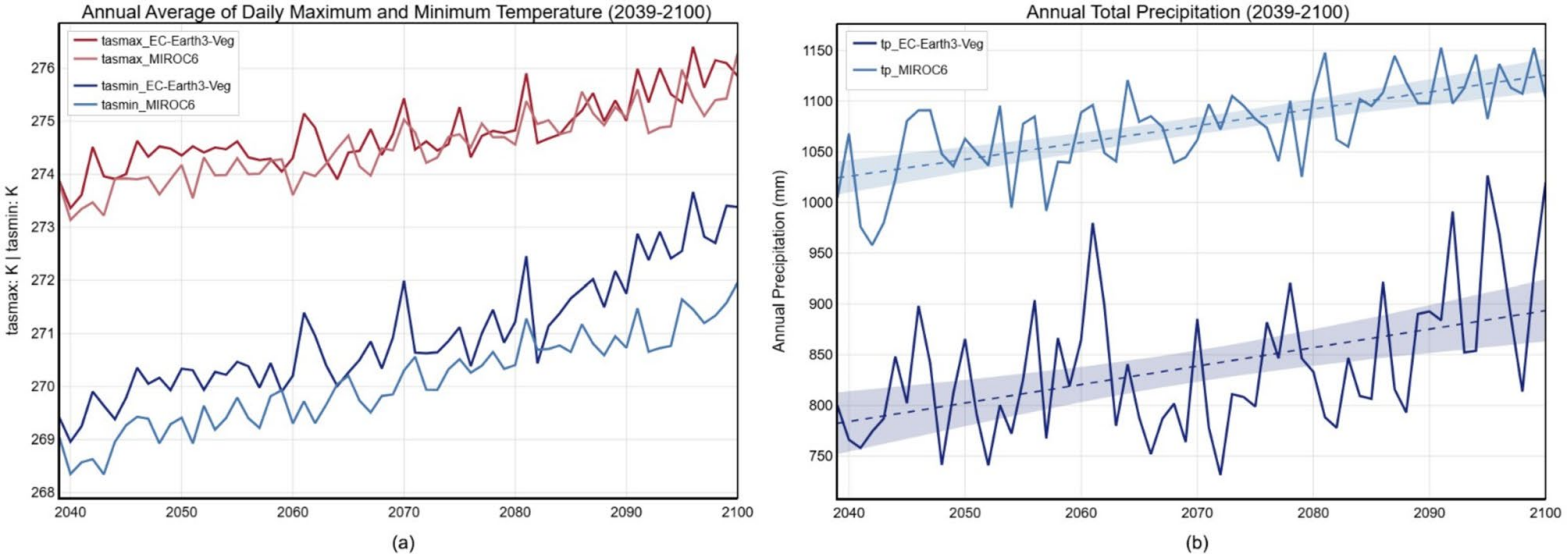
Line chart of average maximum and minimum temperatures from 2039 to 2100 (a), line chart of total precipitation from 2039 to 2100 (b).

As demonstrated in (Fig. 6a), both the warm-wet and hot-dry scenarios indicate an increased trend in the annual mean maximum and minimum temperatures within the study area. The hot-dry model under the SSP5-8.5 scenario anticipates the most rapid warming rate, whereas the warm-wet model projects a little slower rate. The graphs for both variables exhibit clear interannual changes while consistently trending upward despite these variations. The rate of increase in Tmin exceeds that of Tmax, leading to a reduced diurnal temperature range.

Trends in precipitation changes exhibit distinct model reliance. Figure 6b illustrates time series analysis that reveal significant rising trends in annual total precipitation from 2039 to 2100. The MIROC6 model indicates that annual total precipitation peaks at 1152.66 mm. Despite the presence of long-term trends, both models demonstrate significant interannual variability. The EC-Earth3-Veg model outcomes indicate intervals of persistently low precipitation from 2065 to 2089, juxtaposed with an exceptionally high amount of 1026.77 mm in 2096.

### Spatial patterns of future climate changes

**Fig. 7:**
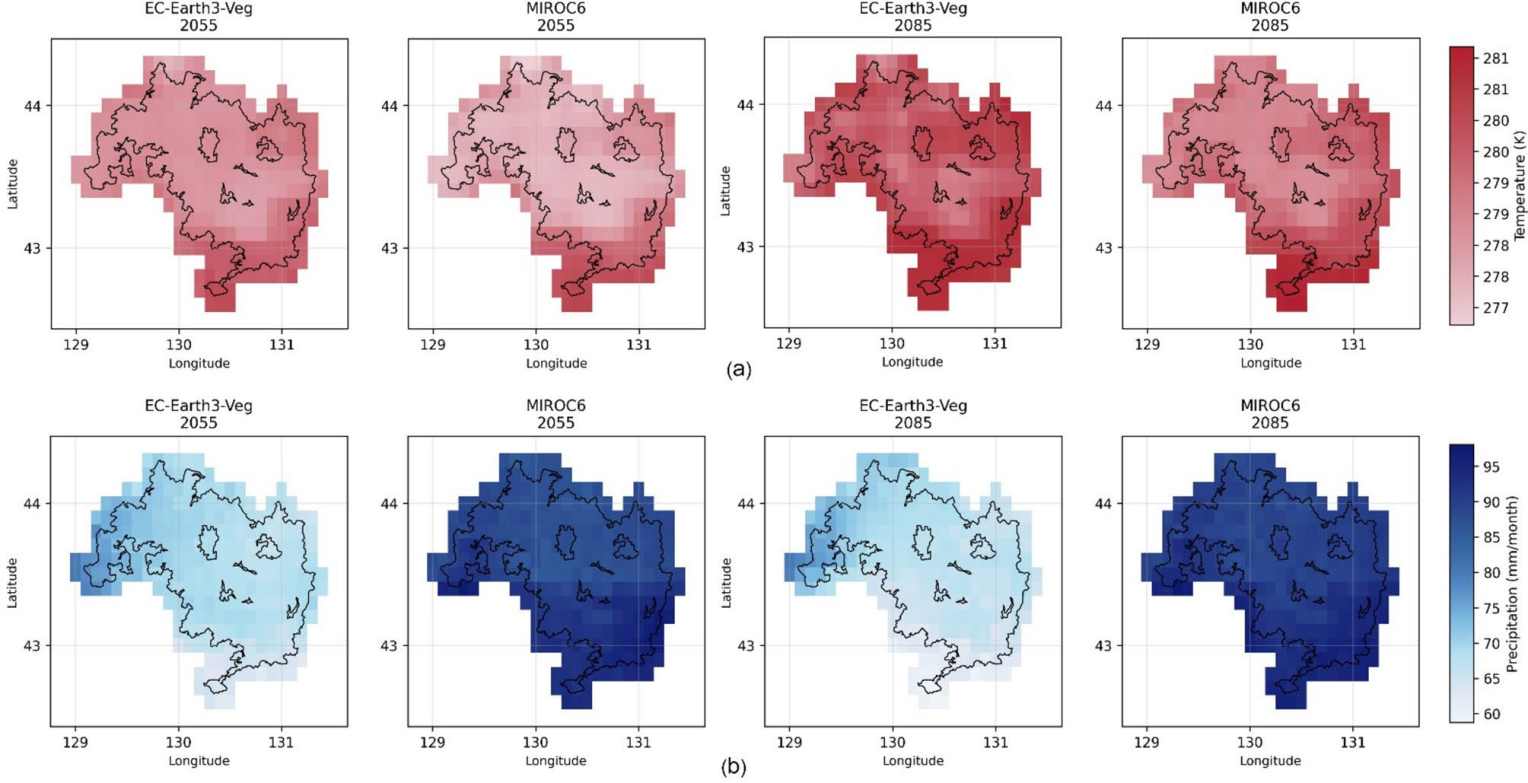
Maps of annual mean temperature and monthly mean total precipitation from the EC-Earth3 and MIROC6 models for 2055 and 2085.

The spatial distribution of future annual mean temperature in the research area (Fig. 7a) retains the latitudinally influenced characteristic of higher temperatures in the south and lower temperatures in the north, as observed in the reference period (Fig. 3). In both the hot-dry and warm-wet scenarios, the geographical temperature distributions for the mid-century (2055) and late-century (2085) periods are predominantly aligned with the reference period. The EC-Earth3-Veg model, which forecasts significant warming, predicts that annual mean temperatures would attain 278.55 K by mid-century and 280.00 K at century’s end, demonstrating a marked rising trajectory. This indicates that future warming results in a general increase in the baseline throughout the region, rather than altering local patterns.

Unlike temperature, the regional patterns of future monthly mean total precipitation will see substantial alterations (Fig. 7b). In the reference period (Fig. 3), precipitation displays a saddle-shaped distribution, characterized by elevated values on the eastern and western peripheries and diminished values in the middle river basins, presumably due to orographic lifting effects from the adjacent mountains. In the hot-dry scenario, this pattern will transition to a longitude-dependent arrangement marked by increased precipitation in the west and decreased precipitation in the east (Fig. 7b). In contrast, within the warm-wet scenario, the geographical pattern of the reference period becomes slightly indistinct but does not completely vanish, as future precipitation trends towards increased spatial uniformity (Fig. 7b). The sharply divergent estimates highlight significant uncertainties in future moisture transport routes and precipitation processes in the region.

### Assessment of future extreme climate events

**Fig. 8:**
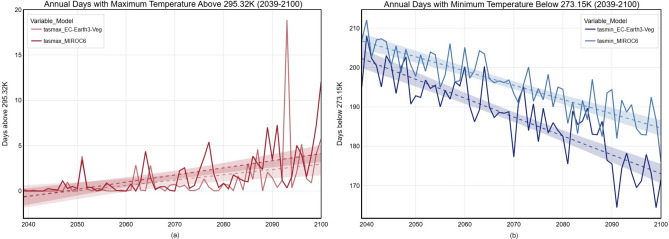
Number of days each year exceeding the maximum temperature threshold (295.32K) (a) and number of days each year below freezing (b), as well as linear regression of the trends and 95% confidence intervals.

Figure 8a illustrates that the annual mean number of extreme heat days, defined by the 99th percentile of reference period data as the threshold for daily maximum temperature exceeding 295.32 K, demonstrates a distinct linear increasing trend across both climate scenarios. The “warm-wet” model indicates a rapid growth trend with a slope of 0.07 days/year, exhibiting high certainty (R^2^ = 0.37) and achieving statistical significance at a very high level (*p* < 0.001). The hot-dry model forecasts elevated annual mean temperatures; however, its growth rate for extreme heat days is marginally lower at 0.06 days/year, accounting for 15% of the data variance (R^2^ = 0.15) and exhibiting high statistical significance (*p* = 0.001). Simultaneously, as shown in Fig. 8b, the annual mean number of frost days (daily minimum temperature < 273.15 K) exhibits a linear decreasing trend. In the hot-dry model, the reduction in frost days occurs at a rate of 0.5 days annually, with this linear trend explaining 73% of the variability in the data (R^2^ = 0.75) and demonstrating a high level of statistical significance (*p* < 0.001). The warm-wet model demonstrates a notable declining trend of 0.47 days per year, exhibiting an explanatory power of 73% (R^2^ = 0.73) and a high level of statistical significance (*p* < 0.001.

### Frequency and intensity of extreme precipitation events

**Fig. 9:**
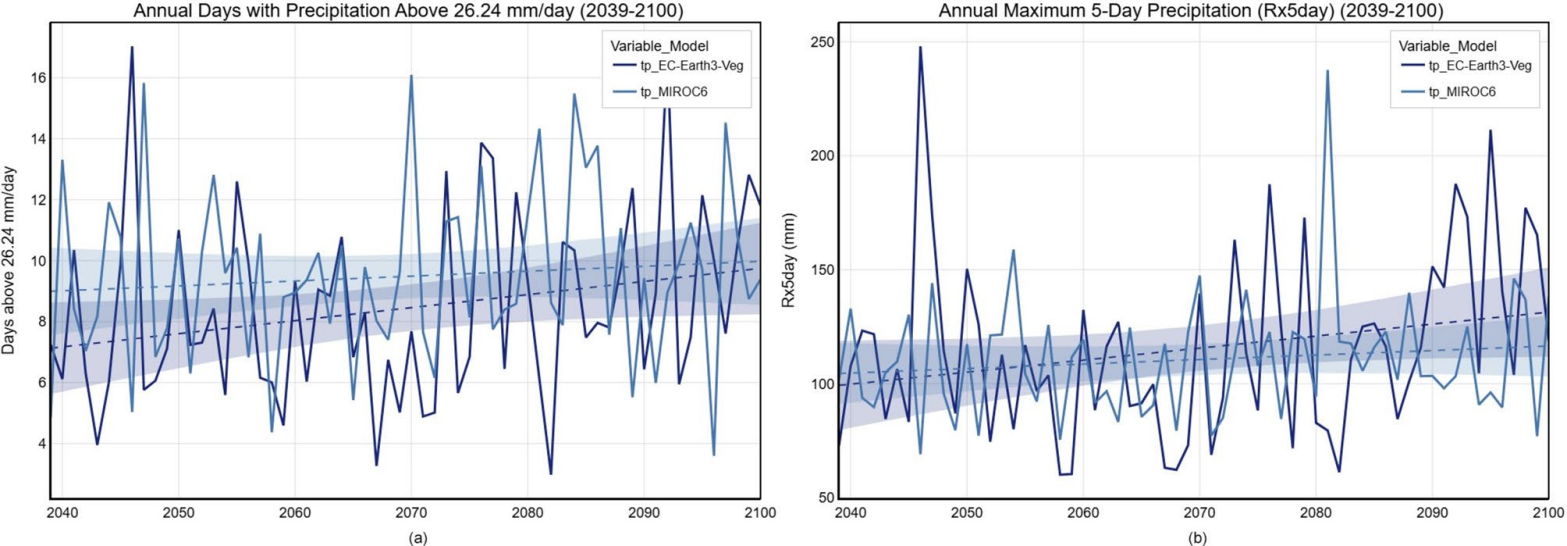
Number of days each year exceeding the 99th percentile value of the reference period (2000-2014) (a),maximum 5-day consecutive precipitation (b).

Figure 9a illustrates projections of the annual mean number of heavy precipitation days (daily precipitation > 295.32 K), indicating significantly divergent evolutionary pathways between the two models. The warm-wet model predicts a greater baseline frequency of heavy precipitation events, indicated by its higher intercept of 1096.9 compared to 916.7. However, the long-term growth trend is not statistically significant, exhibiting a linear slope of merely 1.27 days/year (*p* = 0.25). The linear trend explains only 2% of the data variability (R^2^ = 0.02), suggesting that significant interannual fluctuations obscure the long-term trend. The hot-dry model indicates a lower baseline annual mean of heavy precipitation days; however, its growth trend is statistically significant, exhibiting a linear increase of 4.13 days per year, which meets the 99.9% confidence threshold (*p* < 0.001). This trend accounts for roughly 20% of the variance in the data (R^2^ = 0.17), indicating a significant increase in the occurrence of heavy precipitation events in this scenario.

Analyses utilizing the annual maximum consecutive 5-day precipitation (RX5day) as an indicator reveal comparable dynamics concerning the intensity of extreme precipitation, as illustrated in Fig. 9b. Both models do not demonstrate a statistically significant long-term growth trend in RX5day. The hot-dry model exhibits a notable upward trend at a rate of 0.47 mm/year, demonstrating statistical significance (*p* < 0.001) and a 95% confidence interval of [− 0.07, 1.00] mm/year, which encompasses zero. The warm-wet model exhibits a modest upward trend of 0.20 mm/year, which is not statistically significant (*p* = 0.19), and has a 95% confidence interval of [− 0.13, 0.67] mm/year. In summary, while the trends lack statistical significance, the hot-dry model indicates a higher potential for increased intensity of extreme precipitation in the future.

### Climate risk assessment

#### Risks to the survival of key species

In the hot-dry scenario, climate risks directly threaten the survival chains of key species. Projected elevated temperatures and drought conditions are expected to reduce habitat suitability in core areas for Amur tigers and leopards within the park, such as the Hunchun sector. Reduced canopy cover of keystone tree species, such as Korean pine and linden, may subsequently affect the population densities of primary prey, including wild boar and roe deer. This situation could compel tigers to expand their foraging ranges and increase energy expenditure, ultimately compromising adult tiger survival rates. Moreover, the decrease in water sources along transboundary migration corridors, such as the Sikhote-Alin connection at the China-Russia border, poses a direct threat to the survival of less resilient cubs during migration. Population viability analysis for the Changbai-Primorye tiger subpopulation (~ 38 individuals) projects a baseline extinction probability exceeding 64% within 100 years even without additional climate-driven stressors, rising to 99.8% under severe inbreeding conditions^30^. Prey depletion represents the proximate mechanism: ungulate richness accounts for 40.2% of the predictive contribution to tiger distribution models in Northeast China, and habitat suitability for the primary prey species^31^, wild boar and roe deer, is strongly tied to broadleaf forest productivity and soil moisture conditions that are directly degraded under sustained drought.

In the warm-wet scenario, risks predominantly present as physical disruptions affecting reproduction and population connectivity. Extreme precipitation events are expected to increase landslide risks in rocky den sites favored by Amur leopards, primarily located in low-elevation hills. The destruction of these dens presents direct threats to cub survival during the breeding season, which occurs annually from March to May. Intense rainfall may hinder fruiting in understory shrubs, reducing food availability for prey like hares and indirectly disrupting the food chain of leopards. Spatially explicit corridor modeling identifies the Laoyeling-Dalongling area as the primary connectivity zone between NTLNP and the southwestern Primorye tiger population in Russia^31^. Population simulation results demonstrate that habitat connectivity, specifically dispersal exchange at historical rates of ~ 0.22% between the two populations, represents the only single management intervention capable of generating a positive tiger population growth rate,without it, extinction probability exceeds 64% within a century. The near ten-fold increase in extreme precipitation frequency projected under the warm-wet scenario therefore does not merely represent an infrastructure risk, but a demographic threshold concern for the population’s long-term viability^30^.

#### Risks to core functions

In the hot-dry scenario, arboreal forests within the park’s western transboundary corridors, such as the Dongning sector, may experience fragmentation zones. This could enhance ecological resistance to tiger dispersal across regions and diminish corridor connectivity indices, thereby potentially impeding genetic exchange between Chinese and Russian populations. Additionally, increased outbreaks of pests and diseases, such as the oak longhorn beetle, in Mongolian oak forests may lead to higher tree mortality rates and reduce the capacity for carbon sequestration in these forests.

In the warm-wet scenario, gully erosion caused by extreme precipitation may compromise patrol roads in eastern corridors, such as the Hunchun-Russia’s Khasan district, thereby complicating human monitoring and anti-poaching efforts. This may worsen soil erosion in understory layers, hindering forest regeneration and consequently degrading the hidden habitats critical for the survival of tiger and leopard cubs.

## Discussion

The discussion section of this study initiates with the formulation of a “risk envelope” to systematically disclose and delineate the “compound risks” confronting the Northeast Tiger and Leopard National Park. The research establishes a high-resolution, high-fidelity uncertainty envelope for contrasting climate scenarios, delineated by two extreme models from the CMIP6 ensemble: (1) the “dry-hot” boundary, exemplified by the EC-Earth3-Veg model, which demonstrates the greatest future temperature rise (max ΔT) under the SSP5-8.5 scenario; (2) the “warm-wet” boundary, represented by the MIROC6 model, which indicates the highest future precipitation increase (max ΔP). This offers park managers a “stress test” framework: the actual future regional climatic trajectory, irrespective of its precise form, is extremely probable to reside inside the multidimensional possibilities space defined by these severe limits. Consequently, effective management techniques must concurrently tackle the dangers posed by these limits. This study quantifies extreme events and uncovers a critical scientific result essential for management strategies: a significant compound risk. This finding is counterintuitive because the EC-Earth3-Veg model, designated as the “dry-hot” boundary due to its maximum ΔT, exhibits a more pronounced and rapid statistical growth trend in the frequency (Fig. 9a, *p* < 0.001) and intensity (Fig. 9b, *p* < 0.001) of extreme precipitation events than the “warm-wet” boundary model (MIROC6). This phenomenon serves as a significant warning of prospective risks: it suggests that the physical mechanisms responsible for exceptionally high temperatures in the region do not exclude the possibility of excessive precipitation. If managers concentrate exclusively on the average condition of the EC-Earth3-Veg model (Fig. 7b) and emphasize drought mitigation based on its “dry-hot” attributes, they will neglect the possible risk of “flash flood disasters” in this context. This study posits that the primary concern for the Northeast Tiger and Leopard National Park is not merely isolated “dry” or “wet” threats, but a compounded threat c characterize as “flash flood on drought”, which entails unexpected intense rainfall occurring amongst elevated temperatures and drought conditions. This necessitates management solutions that possess dual resilience to guarantee the ecosystem’s long-term sustainability.

### Forward-looking management strategies based on “compound risks”

The quantified results of this study, particularly the identification of “compound risks”, offer a vital scientific foundation for developing effective and proactive management techniques that may concurrently tackle numerous threats.

1. All scenarios indicate a substantial rise in temperature (Fig. 4) and a swift reduction in frost days (R^2^ = 0.73–0.75; Fig. 8b). This necessitates a transition from static fire risk prevention to a “dynamic bio-climatic” risk management strategy. This alteration signifies an increased fire hazard in the “dry-hot” situation and suggests that the decrease in frost days may result in a heightened overwintering survival rate of pests, such as pine caterpillars. Consequently, management strategies must transcend conventional static fire risk assessments to establish an integrated dynamic fire risk early warning system that includes extreme high temperature forecasts (Fig. 8a) and concurrently implement a biological disaster monitoring network to facilitate coordinated prevention of bio-climatic coupled risks.
2. The regional distribution of future precipitation will experience a significant alteration, transitioning from the historical “saddle-shaped” configuration to a new pattern, whereas the incidence of extreme precipitation events will escalate most swiftly under the “dry-hot” scenario. This necessitates the fortification of natural corridors and infrastructure through “pattern reconfiguration” and “extreme frequency augmentation”. The study findings suggest that biological corridors and infrastructure, including culverts and bridges, constructed according to historical precipitation patterns are at danger of functional incompatibility under the new climate regime. The abrupt intense precipitation in the “dry-hot” scenario (Fig. 9a) will markedly elevate the likelihood of initiating geological calamities. Management strategies ought to utilise the extreme precipitation information from this study (Fig. 9) to perform systematic “stress tests” on all critical corridors and facilities, subsequently implementing adaptive retrofitting and redundancy design as necessary.
3. The increase in the temperature baseline (Fig. 8a) and the “drought and flood compound” risk (Fig. 7a) need a reassessment of the siting rationale for climate refugia and the establishment of a “dual-protection” climate refugia network. Regions capable of mitigating excessive high temperatures (Fig. 7a) should not be situated in prospective high-risk areas for extreme precipitation (Fig. 7b). Management strategies must prioritize the preservation and restoration of critical habitats with significant temperature regulation and flood resilience, such as water source conservation forests, to establish a refugia network capable of enduring compounded climatic stress.

By comparing the quantitative results of this study with published research on climate change in Northeast Asia and Northeast China, it can be observed that the overall trends revealed in this study are completely within the general range of changes in the region, but exhibit unique characteristics in local extremes. In terms of warming amplitude, the significant warming trend reflected by the ‘dry-hot’ boundary (EC-Earth3-Veg) set in this study is highly consistent with the regional consensus under the CMIP5/CMIP6 framework that Northeast China is a high-latitude warming hotspot. Under the global warming thresholds of 1.5 °C and 2.0 °C, the warming in Northeast China will reach 1.75 ~ 1.88 °C, with an additional 0.74 ~ 0.76 °C increase under the 2.0 °C threshold; meanwhile, CMIP6 models generally predict 0.2 ~ 0.3 °C higher warming than CMIP5 in this region^32^. The intense warming captured in this study under the SSP5-8.5 scenario confirms this strong regional warming signal. Regarding extreme precipitation, this study found a surge in the frequency and intensity of extreme precipitation under the ‘dry-hot’ boundary, which aligns with the general pattern of significant increases in regional extreme precipitation indices such as RX1day and R95p. Relevant studies indicate that at the national level, the R95p and R99p indices will increase by 11.0–11.9% and 21.6–22.4%^33^, respectively, and under the CMIP6 framework, the increase in heavy precipitation events could reach 40% and 70% at global warming levels of 2 °C and 3 °C. This study further systematizes the compound risk characteristics of coexisting drought and extreme wet events based on this quantification. Although the evolution trends are consistent, the extreme numerical fluctuations in this study are often higher than the aforementioned regional average levels. These numerical differences are not scientific contradictions but are the result of extreme boundary model selection, providing national parks with more precise, ‘bottom-line thinking’-oriented risk warnings.

Under the ‘dry-heat’ boundary in EC-Earth3-Veg, the frequency and intensity of extreme precipitation increase significantly (*p* < 0.001). This counterintuitive phenomenon is essentially the result of the coupling between thermodynamic intensification and dynamic circulation anomalies. The thermodynamic effect lays the physical foundation through the Clausius-Clapeyron (C–C) relationship. For every 1 °C rise in temperature, the atmosphere’s moisture-holding capacity increases by about 7%. In a dry-heat context, strong surface sensible heat flux leads to an elevation of planetary boundary layer height and a concentration of convective available potential energy (CAPE). Although this thermodynamic environment limits regular precipitation, it provides abundant energy for explosive triggering of convective activity, making the intensity of individual precipitation events more explosive^34^. It also drives key moisture transport through dynamic mechanisms. The risk of “sudden floods during drought” in Northeast China is closely related to the anomalous northward rise of the Western Pacific Subtropical High (WPSH) and the Okhotsk Sea blocking high. The westward extension and northward rise of the subtropical high create a strong channel for transporting warm and moist air to Northeast China. When abundant moisture enters a highly unstable atmosphere driven by dry-heat conditions, it is very likely to trigger instantaneous strong convective systems. This dual intensification mechanism indicates that under a dry-heat background, by increasing the atmosphere’s moisture-carrying capacity and circulation instability, the frequency of extreme convective precipitation is actually exacerbated, forming a severe compound risk^35^.

### Limitations and future prospects of the study

This study’s “contrasting climate futures” uncertainty envelope serves primarily as a stress-testing instrument, with its principal value in delineating acceptable risk bounds rather than offering probabilistic scenario forecasts. This paradigm intentionally refrains from attributing probabilities to future paths, therefore evading the subjective uncertainties arising from model weight distribution.

The results of this study—especially the unusual enhancement of extreme precipitation occurrences by the EC-Earth3-Veg model under the “dry-hot” boundary—offer a definitive pathway for further exploration of physical mechanisms. Future research may enhance this framework by including Bayesian Model Averaging (BMA) or alternative ensemble techniques to quantify conditional probability densities for various situations, thus augmenting decision-making information layers for managers with probability weighting. Furthermore, the present study’s ecological risk assessment relies on qualitative inference from projected climate variables rather than quantitative coupling with species distribution or population dynamics models^36^. Future work integrating the climate projections developed here with ensemble habitat suitability modeling or population viability analysis tools would enable direct translation of extreme climate indices into population-level extinction probabilities, providing a more rigorous quantitative foundation for adaptive conservation planning^33^.

This study systematically accomplished three interconnected core objectives: (1) the development of a high-resolution, high-fidelity uncertainty envelope for contrasting climate futures; (2) within this framework, the quantification of extreme climate risks for the Northeast Tiger and Leopard National Park, including the critical identification of the paradoxical compound risk of “flash flood on drought”; (3) and the subsequent proposal of proactive management strategies based on thorough risk characterization, encompassing dynamic bio-climatic risk early warning systems, extreme scenario stress testing of infrastructure, and a climate refugia network with dual resilience to elevated temperatures and flooding.

These contributions collectively illustrate that adaption systems reliant only on singular mean climate projections possess inherent structural deficiencies. Only resilient designs grounded on the “risk envelope” rather than average trends can guarantee the long-term durability of this premier socio-ecological system against multifaceted climate stressors.

## Data Availability

The datasets generated and analyzed during the current study are not publicly available due to ongoing subsequent research, but are available from the corresponding author on reasonable request.
